# Capacity and needs assessment of veterinary services in Vietnam in biosecurity, biosafety and One Health

**DOI:** 10.1371/journal.pone.0295898

**Published:** 2024-01-11

**Authors:** Aashima Auplish, Thi Thu Tra Vu, Phuc Pham Duc, Alexandra Green, Harish Tiwari, Tambri Housen, Mark Anthony Stevenson, Navneet Dhand

**Affiliations:** 1 Sydney School of Veterinary Science, The University of Sydney, Sydney, NSW, Australia; 2 Faculty of Veterinary Medicine, Vietnam National University of Agriculture, Hanoi, Vietnam; 3 Institute of Environmental Health and Sustainable Development, Hanoi, Vietnam; 4 Center for Public Health and Ecosystem Research (CENPHER), Hanoi University of Public Health, Hanoi, Vietnam; 5 National Centre for Epidemiology and Population Health, Research School of Population Health, The Australian National University, Canberra, ACT, Australia; 6 Faculty of Veterinary and Agricultural Sciences, The University of Melbourne, Parkville, Victoria, Australia; Federal University of Technology Minna, NIGERIA

## Abstract

The Asia-Pacific region is recognised as an epicentre of emerging infectious diseases (EIDs), of which 75% are zoonotic in nature. Vietnam is recognised as a potential hotspot for zoonotic EIDs. There is a growing recognition that progress towards global health security requires greater focus on collaboration between the human health and animal health sectors to control diseases at their animal source and prevent against human health impacts. Assessment of veterinary epidemiology capacity in Vietnam is paramount to strengthening the health security of Asia-Pacific. This study aims to evaluate the national capacity and needs of veterinary services in Vietnam in biosecurity, biosafety and One Health. A cross-sectional, convergent mixed-methods study was conducted between November 2020 and April 2021. An online questionnaire was administered to government-employed field veterinarians. Descriptive analyses and logistic regression models were performed using survey responses to understand capacity in the field. Semi-structured interviews were also conducted with stakeholders in veterinary services including government, academia, research institutes, non-profit and international organisations. Coding and thematic analysis using a deductive approach was used for data collected from interviews to contextualise findings from the survey and understand institutional capacity. In total, 178 field veterinarians completed the online survey and 25 stakeholders were interviewed. The majority of participants had reported receiving training in biosecurity and biosafety, including use of personal protective equipment. Most respondents reported practicing good biosecurity measures (92%) and good biosafety measures (88%). Physical and socioeconomic barriers to practicing biosecurity were reported to be prevalent for smallholder farmers, which may suggest a gap in the capacity of veterinary services to provide cost-effective and practical biosecurity strategies. Seventy five percent of participants had never or rarely participated in One Health approaches in the field in the last 12 months and 69% reported further training as a high priority. There was a knowledge gap reported amongst district and commune-level veterinary staff about the need for, and awareness of multisectoral collaboration. Respondents that completed postgraduate qualifications in epidemiology or Field Epidemiology Training Programs (adjusted OR: 3.06; 95% CI: 1.01, 9.23, p = 0.046) and had longer job tenure between 10–12 years (OR: 10.38; 95% CI: 3.06, 35.15, p = <0.001) were more likely to have higher levels of experience in One Health. This study identified gaps in knowledge, attitudes and adoption of practices related to biosecurity, biosafety and One Health specifically in lower-level or less experienced veterinary staff without further training opportunities in epidemiology. These findings enable prioritisation of training, policy, and planning activities to further enhance the national capacity of veterinary services in Vietnam.

## Introduction

The discourse on global health security has become increasingly urgent with the recent advent of the COVID-19 pandemic. While its original source is still unknown, evidence suggests that COVID-19 is an emerging infectious disease (EID) of probable animal origin [1, 2]. More than 60% of human infectious diseases and 75% of EIDs are zoonotic [3, 4]. Other recent zoonotic EIDs include severe acute respiratory syndrome (SARS), Middle East respiratory syndrome (MERS), Ebola disease virus, Nipah virus and highly pathogenic avian influenza (HPAI) (H5N1). Pathogens of animal origin pose an important and growing threat to both human and animal health, food security, development, and poverty reduction globally.

The Asia-Pacific region is recognised as a hotspot for EIDs, including those with pandemic potential [5]. Population growth and movement, expanding urbanisation, and environmental changes caused by agriculture and livestock production intensification are some of the complex processes that increase human-animal interaction and drive disease emergence or spill-over events in this region [5]. Vietnam is one of nine countries globally that is a potential hotspot for zoonotic diseases and is recognised as an epicentre for EIDs [6]. With a large population of 97.3 million, Vietnam has seen rapid economic growth and poverty reduction following political and economic reforms initiated in the 1990s [7]. Developments in economic policy have seen the emergence of intensive livestock production systems [8], and rapid increases in poultry and pig populations have resulted in some of the highest livestock densities in Southeast Asia [9], a known risk factor for disease emergence and amplification [10]. Conversely, it is estimated that 63% of Vietnam’s population still live in rural areas with livelihoods dependent on agriculture, mainly small-scale farming and livestock husbandry [11]. Illegal animal/animal products trade channels, limited resources, knowledge, and lack of investment in prevention measures (such as biosecurity) among small-scale farmers have also led to the rapid spread of transboundary animal disease (TAD) epidemics such as African swine fever (ASF), lumpy skin disease and HPAI, with devastating socioeconomic losses [12–14]. Further, the indiscriminate use of antibiotics in agriculture supports rising antimicrobial resistance (AMR), raising concerns for global health security [15].

The One Health approach recognises that the health of humans, animals and the environment are closely linked and interdependent. The approach mobilises multiple sectors, disciplines, and communities at varying levels and aims to strengthen systems to prevent disease threats at the human-animal-environment interface, including zoonotic diseases, food safety and AMR [16]. Biosecurity and biosafety measures are essential pillars of global health security by preventing the introduction and ongoing transmission of disease into human and animal populations. Implementation of rigorous biosecurity standards by veterinary services coupled with the capacity to implement a One Health approach for problem solving are fundamental to strengthening capacity to prevent EID and TAD outbreaks, improve animal productivity, ensure food safety, and lower antimicrobial use in livestock.

More than ever, there is a growing recognition that progress towards global health security requires greater focus on collaboration between the human health and animal health sectors to control diseases at their animal source and prevent against human health impacts [17]. Vietnam has made substantial progress towards implementing the One Health approach through establishing initiatives such as the One Health Partnership for Zoonoses, adopting national One Health policies, and advancing One Health research and training [18]. Although higher levels of biosecurity and biosafety are seen within larger-scale intensive farming, barriers to good biosecurity continue to exist, especially for traditional smallholder farms likely due to insufficient incentive to invest or reward for good stewardship [19]. While these initiatives represent a necessary first step towards information sharing and capacity building, there is limited information available regarding the knowledge, attitudes and adoption of these measures and practices at the provincial and sub-provincial level to assess the current capacity of veterinary services in Vietnam.

This study was undertaken as a part of a regional capacity and needs assessment conducted in six countries in the Asia-Pacific region by the Asia Pacific Consortium of Veterinary Epidemiology (APCOVE). Using data from qualitative semi-structured interviews and a quantitative online survey, we aimed to assess the current national capacity of veterinary services in Vietnam in biosecurity, biosafety and One Health competencies. The findings from this study will be used to provide regional context and guide aspects of training required to strengthen the capacity of veterinary services in Vietnam.

## Materials and methods

### Study design

**Fig 1:**
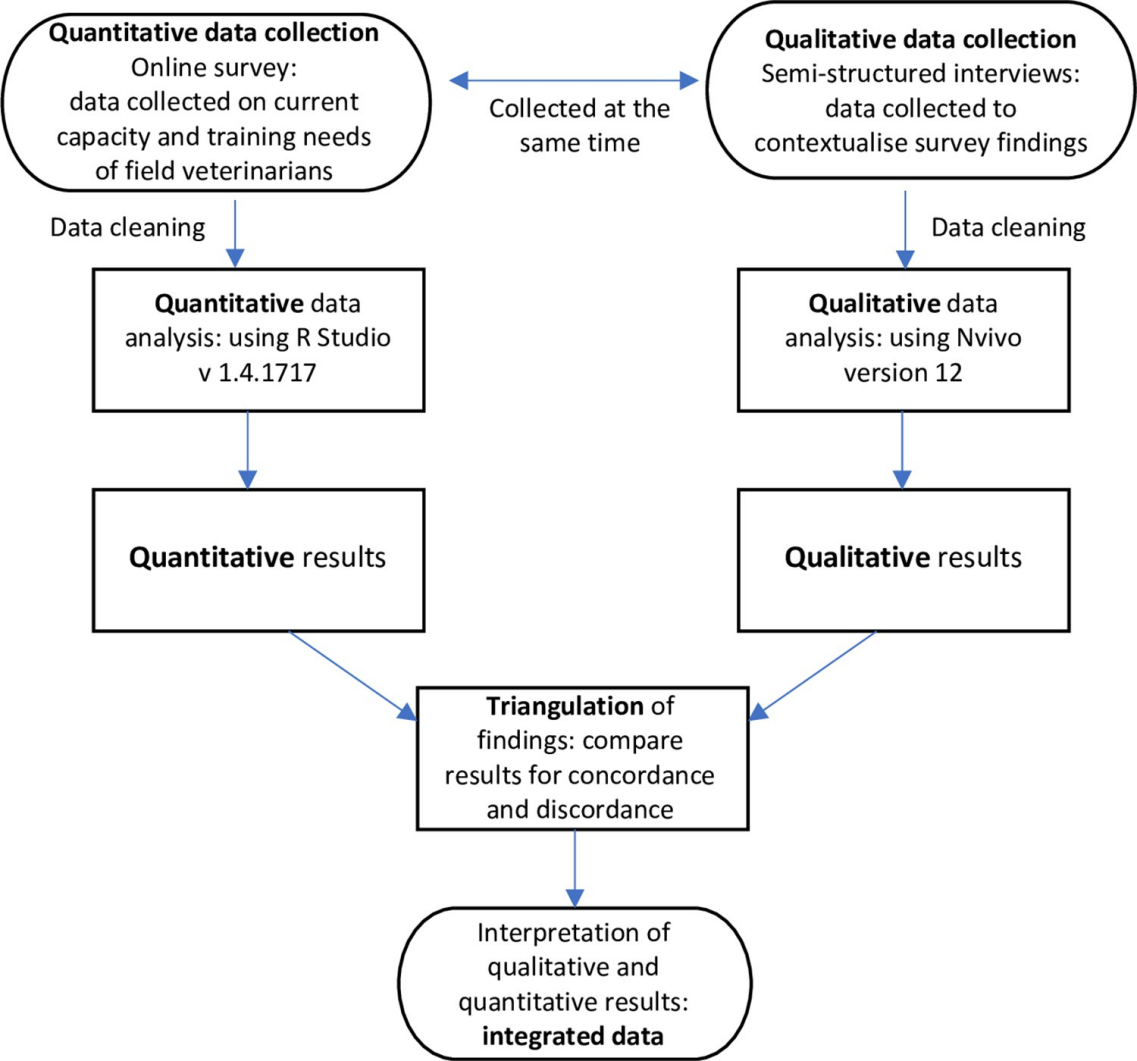
Flow diagram describing convergent mixed-methods study design.

This cross-sectional study used a convergent mixed-methods study design, with quantitative and qualitative data collection and analysis carried out at similar times, followed by an integrated analysis to cross-validate findings (Fig 1) [20]. The quantitative phase used a questionnaire administered online whereas the qualitative phase consisted of semi-structured interviews.

### Study area

This study was conducted in the Socialist Republic of Vietnam, located within Southeast Asia and bordered by China to the north, Laos to the northwest and Cambodia to the southwest. The country comprises eight regions and 63 provinces, which is administratively divided into districts and communes [21]. At the central level, the Department of Animal Health is part of the Ministry of Agriculture and Rural Development (MARD), which has responsibility for seven Regional Animal Health Offices located in seven major regions of the country. Each RAHO corresponds to a region of the country, except for RAHO 1 which covers the Northwest and Red River Delta region of Vietnam. The provincial Sub-Departments of Animal Health (Sub-DAHs) are under the administrative management of the Department of Agriculture and Rural Development and manage District Veterinary Stations, which in turn have links with commune and village veterinary teams [22].

### Participant selection

For the online survey, the study population comprised government-employed field veterinarians (defined as graduates of veterinary science) at the provincial and district level that were contactable via email or instant messaging mobile applications (such as WhatsApp Messenger), based on sampling frame contact lists provided by MARD. For the semi-structured interviews, purposeful sampling was used to identify representatives from the veterinary sector including government and international organisation officials, academia, research institutes and other relevant stakeholders including representatives from the public health, wildlife and environmental sector.

### Ethics statement

Ethics approval for the study was obtained from Human Research Ethics Committee at The University of Sydney (project number: 2020/459). There was no requirement to obtain additional local approval in Vietnam.

Datasets supporting the conclusions of this article are included within the article. Additional data at the level of individual responses is not available as per confidentiality agreements approved by the Human Research Ethics Committee, University of Sydney.

### Quantitative

#### Data collection

The survey questionnaire was self-administered online via the REDCap survey platform [23] and consisted of four open- and 26 closed-ended questions in total. The questionnaire can be found in S1 Appendix. Respondents could also provide additional comments in the form of free-text responses for each closed-ended question. The questionnaire was divided into eight sections, each focusing on a different core epidemiology competency and the respondent’s demographic information. The eight sections focused on: [1] outbreak investigation, [2] animal disease surveillance, [3] data management and analysis, [4] epidemiological surveys and studies, [5] One health, [6] leadership and communication, [7] use of biosafety and biosecurity methods and [8] demographics. In this study, findings from the sections related to One Health, biosecurity, and biosafety are presented. These sections of the questionnaire solicited information about the respondents’ frequency of participation during the past 12 months in One Health practices, biosecurity and biosafety measures in the field, and the priority for further training in each of these core competencies. Findings from other sections will be published elsewhere to allow more in-depth analysis into the results. The questionnaire was developed in English and translated into Vietnamese and piloted by APCOVE members before being administered to participants. The survey was conducted between November 2020 and April 2021. Questionnaire responses were exported from REDCap into Microsoft Excel®. Data were then imported into R Studio version 1.4.1717 [24] (an integrated development environment for R [25] for cleaning and analysis. The map displaying the distribution of survey respondents was developed using GADM [26]. The attempts discontinued by the respondents due to internet, power disruptions or other unavoidable field circumstances were recorded by the REDCap platform as incomplete. Each survey that was started by a respondent was followed-up by one of the co-authors (VTT) to ensure that respondents returned to complete the survey. The truly incomplete survey responses were omitted from subsequent analyses.

Sample size calculations were carried out to determine the number of respondents required to make appropriate inferences from the survey [27]. We estimated that 139 completed surveys were required to be 95% confident that the estimated prevalence of field veterinarians using epidemiological skills related to One Health, biosecurity and biosafety was within 5% of the true population prevalence of 10% (i.e., from 5% to 15%, where ‘population’ refers to field veterinarians in Vietnam).

#### Data analyses

Descriptive analyses were conducted by producing numerical summaries, frequency and contingency tables and graphs. Following this, logistic regression models were fitted. The explanatory variables tested included age (18–34, 35–44, ≥45 years), gender (female, male), work role (district veterinary officer, provincial veterinary officer, other), education level (bachelor, diploma or other, postgraduate), years since graduating from university (<5, 5–9, 10–14, 15–19, ≥20 years), formal epidemiology training completed (no/yes), formal epidemiology workshops attended (no/yes), postgraduate qualification in epidemiology or completion of Field Epidemiology Training Program (no/yes) and job tenure (0–9, 10–12, ≥13 years). The variable of job tenure was categorised by tertiles as it violated the linearity assumption.

Three outcome variables representing the level of experience in: (1) biosecurity, (2) biosafety measures and (3) One Health were created by assigning scores to respondents based on their responses recalling the frequency of participation in activities during the past 12 months (S1, S3 and S4 Tables in S2 Appendix). A score of 0 was given for a response of never or rarely participating and a score of 1 was given for a response of participating about once a month or more than once a month. The total scores for each respondent in One Health, biosecurity and biosafety measures competencies were then counted and subsequently categorised into binary or ordinal outcomes based on the distribution of the scores assigned. For the analyses of level of experience in One Health, a binary outcome variable was used (none-to-low level of experience if the respondent’s total score was 0, and moderate-to-high level of experience if the total score was ≤4) and data were analysed using binary logistic regression models. For biosecurity measures, a binary outcome variable was also used (none-to-low level of experience if the total score was ≤3, vs moderate-to-high level of experience if the total score was 4). For analyses of level of experience in biosafety measures, an ordinal outcome variable was used (low if total score was ≤3, moderate for ≤6 and high level of experience for a total score of 7) and data was analysed using ordinal logistic regression models.

Univariable logistic regression models were fitted to identify associations between explanatory variables and the level of experience in biosecurity, biosafety, and One Health. Explanatory variables with p<0.20 were retained for multivariable analyses. Explanatory variables with >10% of their data missing were excluded from further analyses. A multivariable model for level of experience in One Health was attempted using a manual forward stepwise selection and only those variables with p <0.05 were retained for the final model. Non-significant variables from the stepwise procedure were re-tested in the final model to confirm their non-significance. Potential confounders including age, gender and education level were added to the final models if the parameter estimates of the other variables in the model differed by >20%. Biologically relevant interactions between explanatory variables were tested in the final model and retained only if statistically significant (p<0.05).

### Qualitative

A qualitative approach involving semi-structured interviews with key informants was used. Participants were contacted via email or telephone, with an explanation of the interview process and purpose of the study. All participants agreed to participate in the interview process. Interviews were conducted in Vietnamese either face-to-face or using video teleconferencing software [28] due to movement restrictions posed by the COVID-19 pandemic. All interviews were conducted by bilingual interviewers, VTT and PDP between November 2020 to May 2021 and lasted 60 minutes on average. Verbal consent was provided by each participant before commencing the interview process and audio recording interviews. Due to resource and time constraints, rapid qualitative research methods were employed [29]. The bilingual interviewers listened to interview recordings and familiarised themselves with the data prior to paraphrasing interviewee responses in English. Data collected were deidentified before analysis. The data collected during the interviews were analysed through thematic analysis by HT and AA. After familiarisation with the content of the summarised transcripts, a deductive approach to thematic analysis was used to contextualise findings from the survey. Initial coding and thematic analysis were performed using NVivo software (NVivo version 12, QSR International Pty Ltd). At each stage of the analysis, re-reading the transcripts allowed for reassessment and refinement of the themes developing. Discrepancies were discussed between HT and AA, and further clarified with the interviewer (VTT) to verify that interpretive themes were correct.

## Results

### Sample characteristics

**Fig 2:**
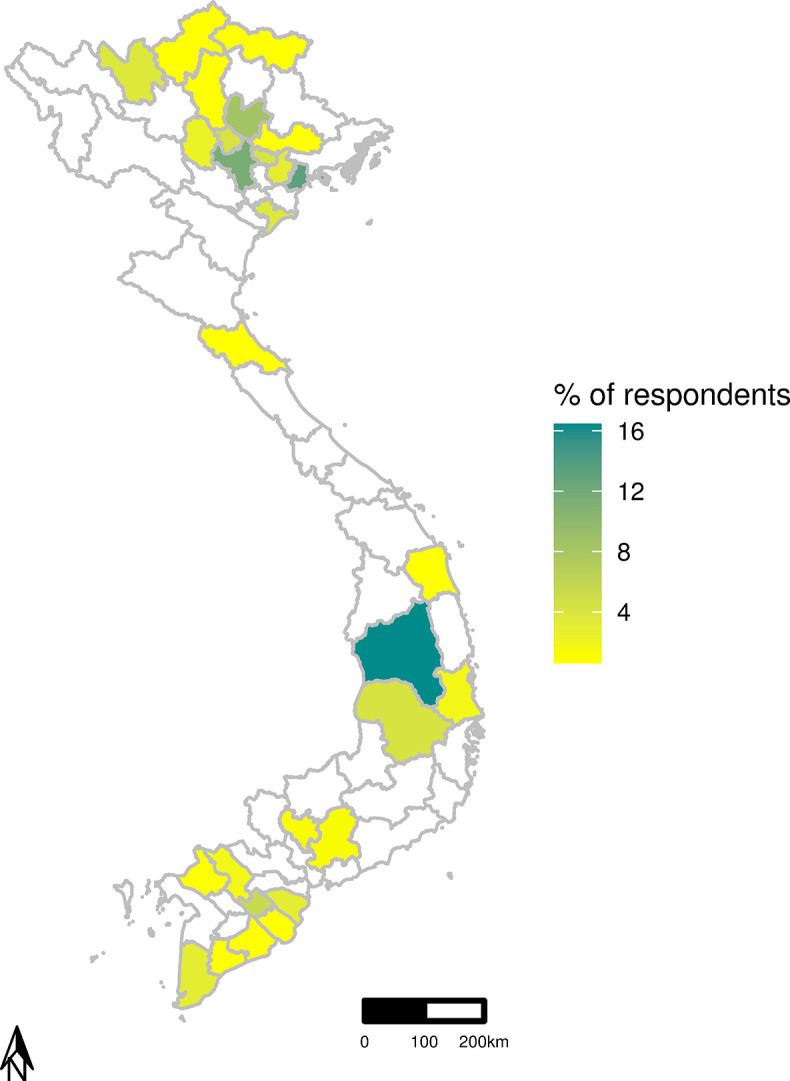
Distribution of survey respondents (field veterinary officers) by province, in the study area of Vietnam. Regions with highest concentration of respondents labelled in black text. The map has been created using GADM data [26]. GADM has provided permission to use this data to create and publish maps under a CC BY 4.0 licence.

A total of 229 survey attempts were recorded by the REDCap platform with 51 truly incomplete entries omitted from subsequent analyses. A total of 178 complete survey responses were included for the quantitative analysis at a level of 97.5% confidence and 5% precision. A total of 25 respondents participated in the interviews. About half (12/25; 48%) of the interviews were conducted face-to-face and the other half (13/25; 52%) using video teleconferencing software [28]. The demographics of survey respondents and interview participants are presented in Table 1. The geographic distribution of survey respondents is displayed in Fig 2.

**Table 1:** Demographic characteristics of online survey respondents (n = 178)* and semi-structured interview participants (n = 25).

Variable	Categories	Survey respondents	Interview participants
		n (%)	n (%)
**Age**	18–34	50 (28.9)	2 (8.0)
	35–44	103 (59.5)	16 (64.0)
	≥45	20 (11.6)	7 (28.0)
**Gender**	Female	92 (53.2)	9 (36.0)
	Male	81 (46.8)	16 (64.0)
**Work role**	District veterinary officer	83 (52.9)	1 (004)
	Provincial veterinary officer	60 (38.2)	17 (68.0)
	Other	14 (8.9)	7 (32.0)
**Education**	Bachelor	107 (63.7)	NA
	Diploma or other	14 (8.3)	NA
	Postgraduate	47 (28.0)	NA
**Years since graduating from university**	<5	16 (10.2)	NA
	5–9	53 (33.8)	NA
	10–14	62 (39.5)	NA
	15–19	18 (11.4)	NA
	≥ 20	8 (5.1)	NA
**Have you completed any formal epidemiology training (outside of your veterinary degree)?**	No formal training completed	76 (42.7)	NA
	Formal training completed	102 (57.3)	NA
	No epidemiology workshops attended	101 (56.7)	NA
	Attended epidemiology workshops	77 (43.3)	NA
	No postgraduate qualification or FETP	155 (87.1)	NA
	Postgraduate qualification or FETP	23 (12.9)	NA
**Job tenure**	0–9	63 (38.4)	NA
	10–12	47 (28.7)	NA
	≥13	54 (32.9)	NA

*Some fields were left blank and may not add to n = 178

### Biosecurity and biosafety

Most participants reported that field veterinary staff are provided training on biosecurity and biosafety measures, including use of personal protective equipment (PPE). Provincial and district level veterinary staff are provided opportunities for more rigorous training and therefore, tend to be more skilled in using biosecurity and biosafety measures. Commune level staff reported limited training opportunities available to them, with exception of the 2003–2006 HPAI outbreak, when training was provided to veterinary staff from all provinces, districts, and communes. However, 81% (n = 138) of respondents still reported a high priority for further training in biosafety and biosecurity (Fig 4). This need for training was echoed within the free-text responses of the survey. Guidelines for biosecurity measures are developed by the DAH for households with livestock and farms when investigating outbreaks. These guidelines and trainings have been produced in collaboration with international organisations such as the FAO.

*“The commune level veterinary staff is not skilled in applying biosafety procedures*. *The district and provincial staff are better skilled*, *with proper training and sufficient equipment*” (*Veterinary academic)*

**Fig 3:**
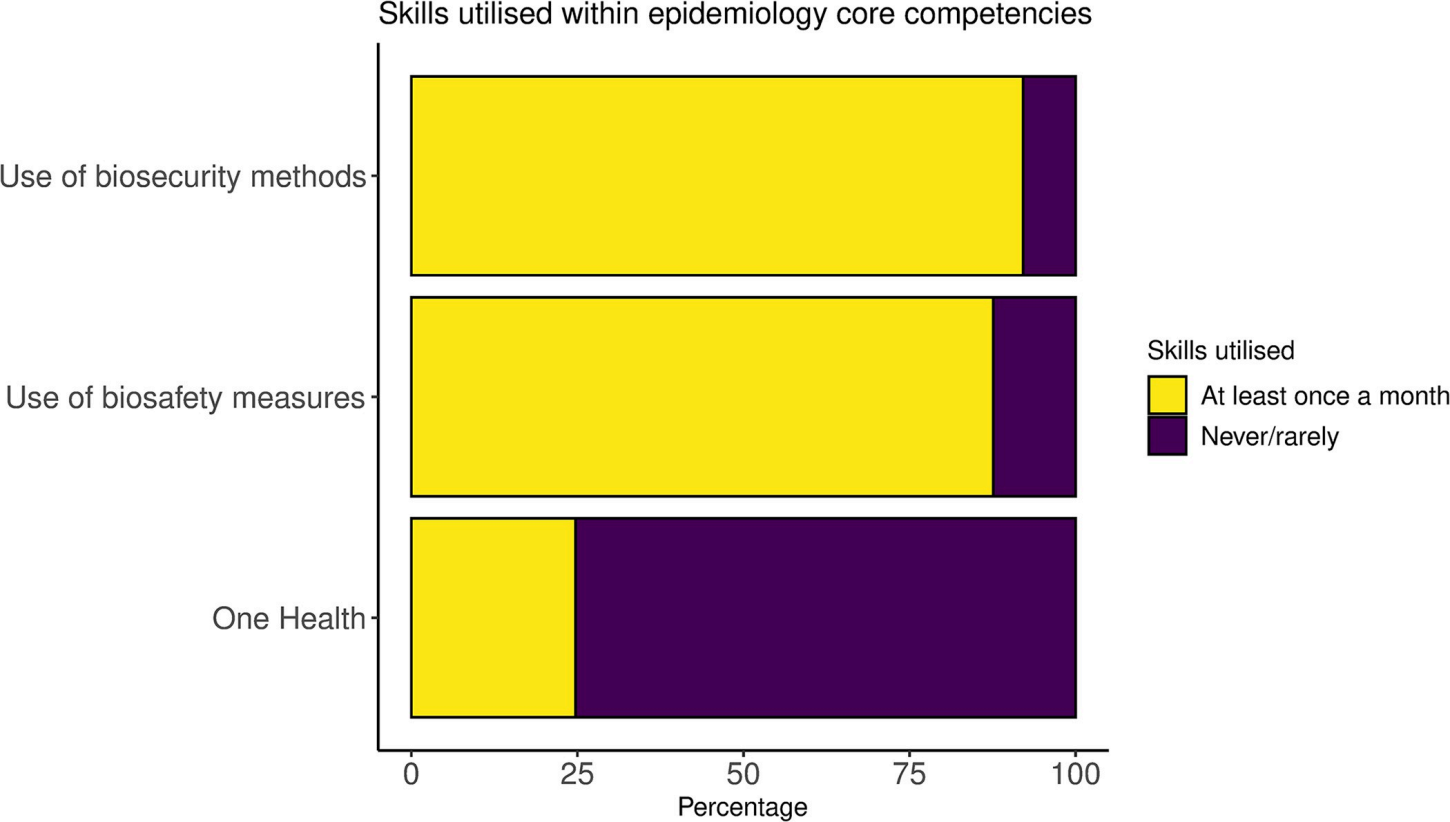
Stacked bar chart showing skills utilised based on scores assigned to respondents for the frequency of participation in One Health practices, biosecurity and biosafety measures during the past 12 months.

Eighty-eight and 92% of respondents reported to practice biosafety and biosecurity measures at least once a month, respectively (Fig 3). Most participants agreed that PPE availability is insufficient, but provincial and district staff are more likely to be well-equipped than commune-level staff, for whom PPE is reported to be scarce. Apart from an overall shortage, other reasons for poor uptake of PPE included the climate, where some staff are reluctant to wear PPE in hot weather conditions. Most survey respondents had used gloves (88%, n = 151), surgical masks (84%, n = 143), gumboots (61%, n = 103) and gowns of overalls (61%, n = 104) more than once a month when handling sick animals, but only 33% (n = 57) had used P2 or N95 respirators (S3 Table in S2 Appendix). Only 57% (n = 96) reported using PPE when disposing of infectious materials. Fifty-nine percent reported visiting farms less than ten times within the last year and only 10% reported visiting farms more than 30 times (Table 2). When visiting farms, most respondents reported using biosecurity measures including the cleaning of boots (94%, n = 158); washing of hands with soap (93%, n = 155); cleaning vehicles (73%, n = 121) and disinfecting equipment (88%, n = 148) before and after visiting a farm (S4 Table in S2 Appendix).

*“There is a lack of PPEs for commune veterinary staff who are working directly with the localities*.” (*Provincial veterinary officer)*

*“When participating in investigation*, *veterinary staff experience a lack of biosecurity equipment*. *In some cases*, *in hot weather*, *veterinary staff are reluctant to wear protective equipment*.” (*Provincial veterinary officer)*

**Table 2:** Number of times farms had been visited by respondents in the last year (n = 131).

Number of times visiting a farm in the last year	n (%)
<10	77 (58.8)
10–20	34 (26.0)
21–30	7 (5.3)
>30	13 (9.9)

Although field veterinary officers at the provincial level and higher reported capability to develop biosecurity protocols for farms, multiple barriers to practicing good biosecurity were reported particularly for smallholders, who predominate farming in Vietnam. Primarily, a concern for the imbalance in the limited number of field veterinary personnel to large number of smallholders was noted. The subsequent inability for training on good biosecurity practices to be organised for all farmers was discussed to contribute towards poor compliance. Other barriers specific to smallholders included limited knowledge about biosecurity and poor access to resources. Physical barriers include having animal barns and/or breeding areas located next to households without establishing clear boundaries and multiple species are often housed together within smaller areas. Additionally, participants reported frequent rushed selling of sick animals and use of salvage feed (leftover food) to feed livestock. On the other hand, larger livestock production systems were reported to have strict biosecurity regulations implemented well.

*“Small farms can only follow a few simple biosecurity procedures*. *Farmers or farm owners have limited knowledge about biosecurity*, *so the application of biosecurity is not thorough…moreover small farms do not have enough resources and funds to ensure biological safety*”. (*Research institute representative)*

*“A number of habits hinders biosecurity effectiveness such as breeding many species together*, *rushed selling when animals get sick*, *free-range livestock*.” (*Veterinary academic)*

#### Predictors of experience in biosecurity or biosafety

After fitting univariable logistic regression models, education level and job tenure were significantly associated with level of experience in biosecurity and only job tenure was significantly associated with level of experience in biosafety at the liberal cut-off of p<0.2. No multivariable models were attempted.

#### One Health

In the context of existing mechanisms enabling cross-sectoral collaboration, the government led initiative, One Health Partnership for Zoonoses was mentioned by participants as a platform bringing together government sectors and other key stakeholders to collaborate on all relevant One Health issues. Participants noted that the joint circular by the Ministry of Health and Ministry of Agriculture and Rural Development (Circular No. 16/2013) provides the legal basis for coordination and collaboration between human and animal health sectors on the prevention and control of zoonotic diseases, including information sharing and communication during surveillance and response. The Joint Circular was the most frequent national One Health policy cited.

*“At the central level*, *the Joint Circular No*. *16 guides the coordination in prevention and control of diseases transmitted from animals to humans (zoonotic diseases*)”. *(Provincial veterinary officer)*

*“…under the Joint Circular 16 for zoonotic disease investigation and respond*, *a mandate between the Ministry of Agriculture and Ministry of Health*, *[there is] information sharing (which is shared on a regular basis at the provincial level) and collaboration to conduct outbreak investigations*, *with a priority on rabies*”. (*International organisation representative)*

**Fig 4:**
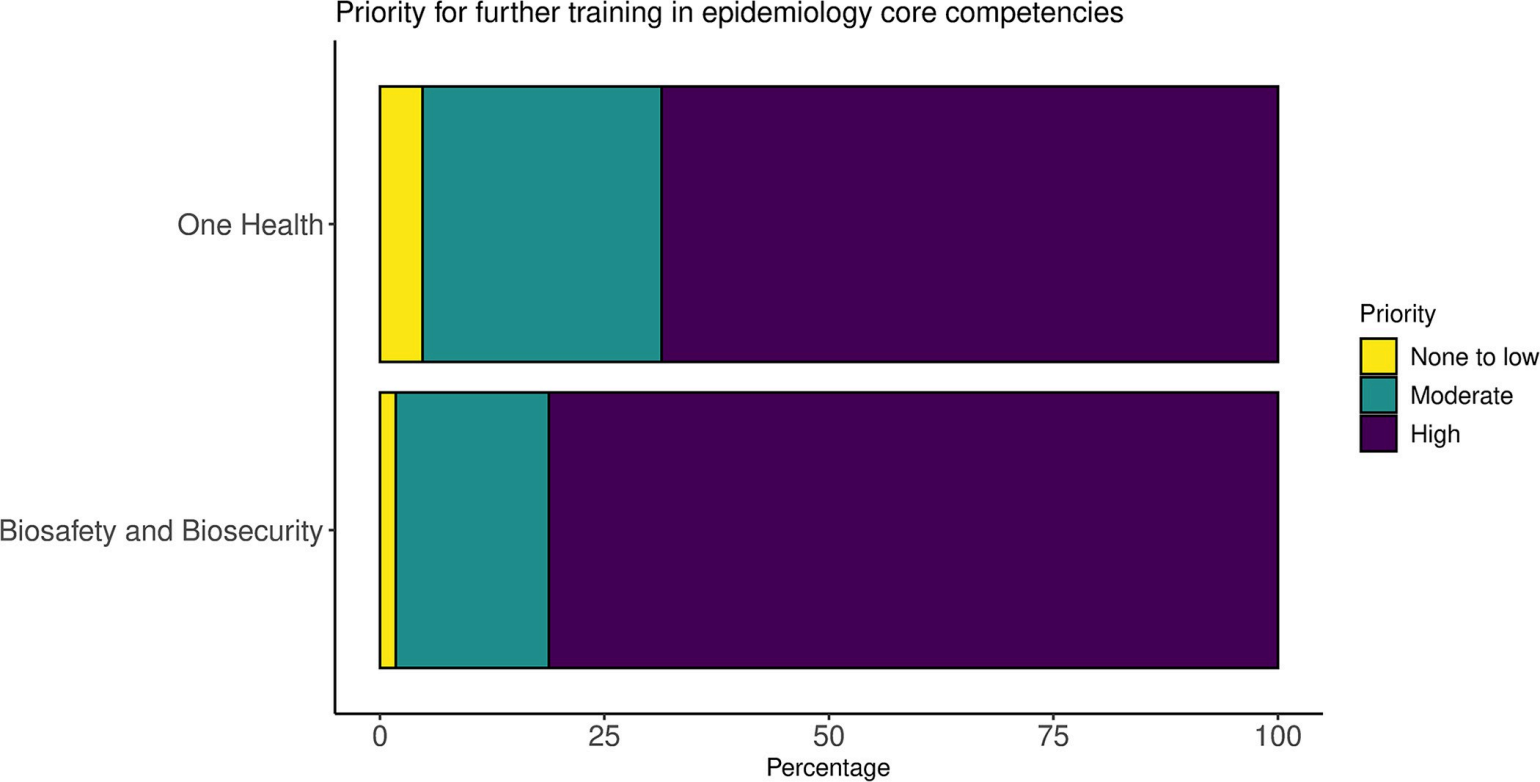
Stacked bar chart showing percentages of respondents reporting a low, moderate or high priority for further biosafety, biosecurity and One Health training.

Overall, 75% of respondents had reported never or rarely participating in One Health practices in the field in the last 12 months (Fig 3). About half (52%, n = 89) of the respondents had rarely assisted in or led the investigation of a zoonotic disease in the past 12 months, and 46% (n = 78) had never been involved in developing a control program for a zoonotic disease. Only 5% (n = 9) had participated in a team involving professionals from animal, human and/or environmental sectors more than once a month (S1 Table in S2 Appendix). Interview participants agreed that there is a knowledge gap, particularly amongst district and commune-level veterinary staff about the need for, and awareness of multisectoral collaboration. Most reported that this may be due to a lack of opportunity, experience, or expertise. Overall, 69% (n = 116) reported a high priority for further training in One Health approaches (Fig 4), which was reiterated in the free-text responses from the survey.

*“…the coordination is only relatively good at the provincial levels*, *while the district and commune levels are still limited*. *[There is a] need to improve the information sharing at district and commune levels*”. (*Provincial veterinary officer)*

Collaboration and communication were reported to occur most effectively at the provincial level (between provincial Centres for Disease Control (CDCs) and Sub-Departments of Animal Health and Livestock Production), but to a limited degree at the district and commune-level. Consultations to evaluate the effectiveness of the implementation of the Joint Circular were suggested to identify gaps so that tailored solutions can be created and implemented. Participants consistently reported that the focus of cross-sectoral collaboration was mainly around avian influenza (AI) and rabies. Surprisingly, AMR was not mentioned, however, it was agreed that proactive One Health measures to enforce food-safety were currently limited.

*“[Collaboration could be improved by] conducting more programs on One Health*, *with more in-depth research and collaboration to promote further understanding of the overlapping and closely relating tasks of animal health and public health*, *raising awareness of the people on zoonoses*, *especially among grassroot veterinary staff and the community*”. (*Provincial veterinary officer)*

*“There is cooperation in prevention and control of zoonotic diseases*, *namely rabies*. *But only when rabies cases occur on humans*, *the two sides will cooperate in investigation*”. (*Provincial-level veterinary epidemiologist)*

Multiple participants reported that funding to support capacity development through the implementation of One Health approaches, such as integrated information systems, is lacking. Lack of funding was also highlighted as a reason for poor sustainability of existing approaches. Participants reported that reporting and information exchange between sectors is currently limited and carried out mostly using printed documents. Strategies like workshops or joint training involving both human and animal health services were suggested to facilitate analysing and improving collaboration between the two sectors to prevent, detect and respond to zoonotic diseases and other health events at the human-animal-environment interface.

*“Currently*, *the cooperation between the two sides remains limited*, *largely on documents*.” (*District veterinary officer)*

#### Predictors of experience in One Health approaches

Following univariable logistic regression analyses, all explanatory variables except gender were eligible for multivariable analyses at the liberal cut-off of p< 0.2. (S2 Table in S2 Appendix). At p<0.05, the variables, completing postgraduate qualifications in epidemiology or Field Epidemiology Training Program (FETP) (adjusted OR 3.06 (1.01, 9.23), p = 0.046) and job tenure were significantly associated with higher levels of experience in One Health approaches after adjusting for education. Respondents with job tenure of 10–12 years (adjusted OR 10.38 (3.06, 35.15), p<0.001) were more likely to have a higher level of experience in One Health when compared to those with a longer or shorter job tenure (Table 3). There were no interactions included in the final model.

**Table 3:** Final binary logistic regression model for level of experience in One Health (n = 178).

Level of experience in One Health				
Variable	None to low	Moderate to high	OR (95% CI)	p-value
**Postgraduate qualification/FETP**				
No	123	32	Ref	0.046
Yes	11	12	3.06 (1.01, 9.23)	
**Years worked in current job**				
0−9^1^	59	4	Ref	<0.001
10–12	29	18	10.38 (3.06, 35.15)	
≥13	32	22	8.58 (2.64, 27.91)	
**Education**				
Bachelor^1^	83	24	Ref	0.15
Diploma and other	12	2	0.46 (0.09, 2.33)	
Postgraduate	29	18	1.99 (0.80, 4.91)	

## Discussion

This study was conducted to understand the current capacity in One Health, biosecurity, and biosafety of veterinary services in Vietnam, identify gaps in knowledge attitudes and adoption of practices on the field. By using this knowledge, we can prioritise aspects of training required for field veterinarians in Vietnam. The findings from this study can be used in conjunction with insights from the World Organisation for Animal Health’s (OIE) Performance of Veterinary Services (PVS) evaluation report [22] and World Health Organization’s (WHO) Joint External Evaluation (JEE) of International Health Regulations (IHR) core capacities [30] to provide a greater understanding of the national capacity of veterinary services.

Currently, Vietnam is engaging in many One Health efforts. The One Health Partnership for Zoonoses is a major mechanism supporting One Health coordination in Vietnam [18, 31], and the Joint Circular (Circular No. 16/2013) provides the legal basis for coordination, information sharing and collaboration between the human and animal sectors in surveillance and response activities [32]. Although participants are aware of the existence of both mechanisms, the results do not indicate how One Health is being carried out in the field. About half of survey respondents had never/rarely assisted in or led the investigation of a zoonotic disease in the past 12 months, and only 5% had participated in a team involving professionals from animal, human and/or environmental sectors more than once a month. This highlights a significant gap in the capacity for application of One Health approaches in the field. The One Health approach encourages information sharing and coordinated responses between sectors that can help improve prevention, early detection, and rapid response to emerging and endemic zoonotic diseases, for which Vietnam is a hotspot. Participants highlighted a knowledge gap particularly amongst district and commune-level staff, which make up majority of field staff surveyed and may contribute towards the poor participation rates. Therefore, it is unsurprising that the majority of field veterinarians surveyed reported a high priority for further training in One Health approaches. An in-depth assessment was carried out by the FAO in 2016 on the implementation of Circular No. 16 in two provinces, identifying similar findings of poor awareness at the district and commune level and a lack of inter-sectoral investigation teams established when a case or outbreak was detected [32]. Further, WHO’s JEE of Vietnam recommended the need for basic and continuing education for field staff and multisectoral training in epidemiology as a priority action to improve the capacity for zoonotic disease prevention, supporting our study findings [30]. Participants suggested workshops as a potential strategy to facilitate analysing and improving cross-sectoral collaboration between animal and human health sectors. An IHR-PVS Pathway National Bridging Workshop program has been launched by WHO and OIE for this purpose, however, Vietnam is yet to participate. The successful collaboration between human health and animal health sectors are likely to depend on better resourcing in the animal health sector, increasing local ownership of the agenda, and ensuring that both sectors can use the full range of regulatory strategies available to achieve objectives [33].

By using level of experience as a proxy indicator for capacity of field veterinarians, our study was able to identify factors associated with higher levels of experience in One Health. Specifically, those who had completed FETP or a postgraduate qualification in epidemiology were more likely to have higher levels of experience. Respondents with job tenure of 10–12 years were also more likely to have higher levels of experience in One Health, which is likely due to greater professional experience, and exposure or opportunity to such approaches. This provides insight into factors, namely training, that may strengthen local capacity. However, due to the type of study design, we cannot distinguish whether high levels of experience lead to opportunities to complete FETP, or FETP completion provides more opportunities to gain experience. Both the FETP and its equivalent for veterinary services, the Applied Veterinary Epidemiology Training, have been established in Vietnam for field veterinarians to participate. The Vietnam One Health University Network (part of the regional Southeast Asia One Health University Network) has developed a field-based joint training course for health and veterinary professionals who are working on infectious disease prevention and control at provincial and district levels [18]. While the mechanisms for One Health training exist, only 15% of survey respondents had completed FETP (13%) or a postgraduate qualification in epidemiology (2%) and of these, only 22% were district veterinary officers. The majority of respondents that had completed further training had participated in epidemiology workshops (76%), which can vary significantly in terms of duration and breadth of learning outcomes, are often not field-based and provide limited opportunity for learning through service. Further emphasis should be put on selection of trainees for training programs to include district-level staff, incentivisation and/or research into barriers that might be preventing district staff from participating. Investment by local government in capacity development at the district and particularly, commune level is necessary. Alongside workforce development, mechanisms for integrated surveillance, diagnostic capacity (in the field and laboratory), information sharing and established joint rapid response teams to facilitate outbreak operations are fundamental to One Health implementation on the field. This will contribute towards improved One Health implementation in the field, specifically timely detection and response capability of frontline workers leading to reduced outbreaks and improved veterinary service provision.

The occurrence and impact of known and novel infectious diseases is likely to increase without adequate biosecurity and biosafety [34]. Good biosecurity and biosafety practices prevent the introduction of disease into human and animal populations and/or limits spread within populations. Livestock production system expansion with increasing consumer demand has been a driver of disease emergence events, exemplified by the global spread of HPAI H5N1. In Vietnam, there have been rapid increases in poultry and pig populations with the emergence of intensive livestock production systems, however, small-scale farms still predominate [11, 35]. The investment in biosecurity for these farmers is often low and poorly coordinated [5] as reported by participants during interviews. Poor biosecurity practices amongst smallholders such as not wearing PPE, inadequate cleaning and disinfection measures, lack of restricting visitors onto properties and isolation or quarantine of sick animals have been reported in other studies within Vietnam [13, 36–38]. Conversely, the majority of survey respondents reported practicing good biosecurity measures when visiting farms, including use of PPE, suggesting the risk of disease introduction is minimised during visits by field veterinarians. It is possible that biosecurity guidelines may be inconsistent with or impractical for management styles of small-scale farms in Vietnam [36], which raises concerns for the ability to prevent and control disease and the subsequent socioeconomic impacts for these farms with increased mortality and reduced productivity of livestock. Improving biosecurity and biosafety practices at the farm level is complex and requires incentivisation (for instance, through government compensation schemes, certification or licensing schemes) given the extra labour and cost that are likely to be incurred [19]. Lack of trust in veterinary services may affect farmers’ decision to implement or improve on-farm biosecurity [13, 39] and the poor adoption of biosecurity measures could also be influenced by the lack of quality veterinary advice and services that farmers receive, as indicated in a previous study carried out with Vietnamese smallholder pig farmers [40]. Eighty one percent of survey respondents reported a high priority for further training in biosecurity and biosafety measures, highlighting an existing gap in capacity. A key focus of this training must be on development of biosecurity strategies for farmers (rather than a one-size-fits-all approach), with a good understanding and awareness of individual farm characteristics and constraints to elicit interest, encourage investment and ensure compliance to good practices [40]. Having veterinary services that can provide cost-effective and practical biosecurity measures that are adapted to each farm is fundamental in the context of Vietnam, where TADs, including zoonotic diseases like HPAI are endemic and have caused significant economic losses to the livestock sector. Assessment of biosecurity practices was limited to farm-level for the purpose of this survey.

### Study strengths and limitations

This study has strengths and limitations. There is limited information available about the current capacity of veterinary services on the ground in the Asia-Pacific region, making this the first study in capturing baseline data. The findings from this study can be used to guide the design of veterinary training programs tailored to the local context and adapted to meet the needs and priorities of field veterinarians in Vietnam. However, self-completed questionnaires come with a risk of recall bias and obsequiousness bias if subjects systematically alter responses in the direction perceived to be desired by the researchers. Commune-level veterinary staff were not captured by the survey. This was in part due to the remoteness of their duty stations and poor access to technology presenting an added difficulty to participation with the movement restrictions in place during the COVID-19 pandemic. Given the gaps identified through the interviews, and as frontline staff, their inclusion in future studies is recommended to ensure a more representative sample. Lastly, future surveys would benefit from asking respondents to rank their priorities for further training to gauge specific competencies which ranked the highest for the majority, as this is likely more useful information to capture and translate into areas of focus for training.

## Conclusion

There is little information currently available about veterinary services on the ground in the Asia-Pacific region. This study provides valuable knowledge about the level of experience in core epidemiology competencies, specifically One Health, biosecurity, and biosafety among field veterinary officers in Vietnam. Survey findings are contextualised when assessed in combination with data collected from key-informant interviews, allowing further insight into the barriers and enablers influencing the current national capacity of veterinary services. We highlight the skills requiring urgent attention through epidemiology training by identifying competencies that field staff are never or rarely participating in. Many of these competencies have also been reported by respondents as requiring a high priority for training. Further investment is required in the operationalisation of One Health approaches in the field and capacity for field veterinary staff to promote good biosecurity and biosafety practices amongst smallholder farmers. Both short and long-term field epidemiology training courses should be coordinated to improve awareness and use of existing intersectoral collaboration mechanisms and competence to deliver biosecurity advice, focusing on the roles and responsibilities that field veterinary officers play. Further research is required to establish the underlying cause and magnitude of differences in training opportunities between central and provincial-level veterinary staff, and district and commune-level veterinary staff. APCOVE is a consortium of veterinary epidemiologists established to strengthen field veterinary epidemiology capacity in the Asia Pacific region, working with government animal health authorities and educators to strengthen their existing on-the-job training programs. The results of this study will ensure that training responds to the needs and priorities of the local animal health workforce. Ultimately, strengthening veterinary services will serve to improve animal and human health outcomes in Vietnam, effectively and sustainably, through enhanced prevention, detection, and response capacity against disease threats and challenges.

## Supporting information

S1 Appendix Questionnaire used for epidemiology capacity and needs assessment online survey. (PDF)

S2 Appendix Additional results from descriptive and univariable analysis. (DOCX)
