# Supplementary material for: Capacity and needs assessment of veterinary services in Vietnam in biosecurity, biosafety and One Health
Source: PLoS One. 2024 Jan 11;19(1):e0295898. doi: 10.1371/journal.pone.0295898 (PMC10783714; doi:10.1371/journal.pone.0295898)
Supplement: S1 Appendix — (PDF) [file pone.0295898.s001.pdf]

Epidemiology Capacity and Needs Assessment Survey

In this survey, we will ask you questions about your experiences and opinions about the need for various epidemiological skills in the field. The survey has the following sections:

- Outbreak investigations
- Animal Disease Surveillance
- Data Management and Analysis
- Epidemiological surveys and studies
- One health
- Leadership and communication
- Use of biosafety and biosecurity methods
- Demographics

Thank you for participating in our survey. Your feedback is important to us.

Outbreak Investigation

1. How often have you done the followings during the last year?

	Never	Rarely	About once a month	More than once a month
Conducted clinical examination for case detection and diagnosis?	◯	◯	◯	◯
Conducted postmortem examination for case detection and diagnosis?	◯	◯	◯	◯
Developed case definitions to classify animals or farms into cases and non-cases?	◯	◯	◯	◯
Applied case definitions to classify animals or farms into cases and non-cases?	◯	◯	◯	◯
Verified that an outbreak is happening?	◯	◯	◯	◯
Conducted trace-forward and trace-backward searches to identify other cases?	◯	◯	◯	◯
Created an outbreak investigation questionnaire?	◯	◯	◯	◯
Collected samples?	◯	◯	◯	◯
Created sample submission forms?	◯	◯	◯	◯
Used sample submission forms?	◯	◯	◯	◯
Transported samples to the laboratory?	◯	◯	◯	◯
Interpreted laboratory results?	◯	◯	◯	◯

Analysed data from an outbreak by space, time and animal group?	◯	◯	◯	◯
Applied preliminary control strategies to contain the outbreak?	◯	◯	◯	◯
Produced an outbreak report?	◯	◯	◯	◯

Any comments:

____

2. How many times have you led or participated in an outbreak investigation in the past 3 years?

- ◯ Never
- ◯ 1 - 2 times
- ◯ 3 - 6 times
- ◯ More than 6 times

3. Based on the skills and experiences of the animal workers in your LGA, what priority would you say there is for further training in conducting outbreak investigations at the moment?

- ◯ No need for further training
- ◯ Low priority
- ◯ Moderate priority
- ◯ High priority
- ◯ Very high priority

Any comments:

____

Animal Disease Surveillance

4. How often have you done the following activities during the last year?

	Never	Rarely	About once a month	More than once a month
Visited farms and talked with farmers to identify possible cases?	○	○	○	○
Followed up reports from informal sources?	○	○	○	○
Reported cases or clusters of cases to the appropriate authorities?	○	○	○	○
Produced a surveillance summary report?	○	○	○	○
Designed a surveillance summary report template that can be used periodically?	○	○	○	○
Designed a surveillance system?	○	○	○	○
Evaluated the operation and disease reporting components of surveillance system?	○	○	○	○
Identified the strengths, limitations and gaps of a surveillance system?	○	○	○	○

Any comments:

____

5. How many times have you led or participated in an animal disease surveillance activity in the past 3 years?

○ Never
○ 1 - 2 times
○ 3 -6 times
○ More than 6 times

6. Based on the skills and experiences of the animal workers in your LGA, what priority would you say there is for further training in animal disease surveillance at the moment?

○ No need for further training
○ Low priority
○ Moderate priority
○ High priority
○ Very high priority

Any comments:

____

Data Management and Analysis

7. How often have you done the followings during the last year?

	Never	Rarely	About once a month	More than once a month
Entered surveillance or outbreak data into a spreadsheet programmes such as MS excel?	◯	◯	◯	◯
Verified surveillance data for data entry errors and typos?	◯	◯	◯	◯
Prepared and interpreted an epidemic curve to describe the outbreak?	◯	◯	◯	◯
Presented surveillance or outbreak data using tables and graphs?	◯	◯	◯	◯
Conducted a statistical test of hypothesis?	◯	◯	◯	◯
Calculated prevalence and incidence measures from surveillance data?	◯	◯	◯	◯
Compared prevalence and incidence between groups to identify risk factors?	◯	◯	◯	◯
Identified trends, patterns, and outliers in surveillance data?	◯	◯	◯	◯
Constructed maps from outbreak or surveillance data?	◯	◯	◯	◯
Identified suspected clusters of disease?	◯	◯	◯	◯

Any comments:

____

8. How many times have you led or participated in data management and analysis activities in the past 3 years?

◯ Never
◯ 1 - 2 times
◯ 3 -6 times
◯ More than 6 times

9. Based on the skills and experiences of the animal workers in your LGA, what priority would you say there is for further training in data management and analysis at the moment?

◯ No need for further training
◯ Low priority
◯ Moderate priority
◯ High priority
◯ Very high priority

Any comments:

____

Epidemiological surveys and studies

10. How often have you done the followings during the last year?

	Never	Rarely	About once a month	More than once a month
Designed a questionnaire for data collection?	◯	◯	◯	◯
Planned and/or conducted a survey?	◯	◯	◯	◯
Planned and/or conducted a cross-sectional study?	◯	◯	◯	◯
Planned and/or conducted a case control study?	◯	◯	◯	◯
Planned and/or conducted a cohort study?	◯	◯	◯	◯
Planned and/or conducted a participatory disease search??	◯	◯	◯	◯
Planned and/or conducted a value chain mapping?	◯	◯	◯	◯
Planned and/or conducted gross margins for a farm?	◯	◯	◯	◯
Planned and/or conducted qualitative risk assessment?	◯	◯	◯	◯
Calculated sample size?	◯	◯	◯	◯
Evaluated a diagnostic test?	◯	◯	◯	◯
Conducted a literature review?	◯	◯	◯	◯

11. How many times have you led or participated in conducting epidemiological surveys or studies in the past 3 years?

- ◯ Never
- ◯ 1 - 2 times
- ◯ 3 -6 times
- ◯ More than 6 times

12. Based on the skills and experiences of the animal workers in your LGA, what priority would you say there is for further training in epidemiological surveys and studies at the moment?

- ◯ No need for further training
- ◯ Low priority
- ◯ Moderate priority
- ◯ High priority
- ◯ Very high priority

Any comments:

____

One Health

'One Health' is a collaborative, multi-sectoral, and trans-disciplinary approach with the goal of achieving optimal health outcomes recognising the interconnection between people, animals, plants, and their shared environment.

13. How often have you done the followings during the last year?

	Never	Rarely	About once a month	More than once a month
Developed a control program for a zoonotic disease?	◯	◯	◯	◯
Assisted in or led the investigation of a zoonotic disease?	◯	◯	◯	◯
Assisted in or led the investigation of a non-zoonotic human disease?	◯	◯	◯	◯
Participated in a team involving professionals from animals, human, and/or environmental sectors?	◯	◯	◯	◯

Any comments:

____

14. How many times have you led or participated in One Health activities in the past 3 years?

◯ Never
◯ 1 - 2 times
◯ 3 -6 times
◯ More than 6 times

15. Based on the skills and experiences of the animal workers in your LGA, what priority would you say there is for further training in One Health at the moment?

◯ No need for further training
◯ Low priority
◯ Moderate priority
◯ High priority
◯ Very high priority

Any comments:

____

Leadership and Communication

16. How often have you done the followings during the last year?

	Never	Rarely	About once a month	More than once a month
Developed educational materials for farmers?	◯	◯	◯	◯
Developed training materials for other animal health workers?	◯	◯	◯	◯
Prepared reports for animal health authorities?	◯	◯	◯	◯
Given an oral presentation?	◯	◯	◯	◯
Prepared a media release?	◯	◯	◯	◯
Given a media interview?	◯	◯	◯	◯
Handled official communication by email?	◯	◯	◯	◯
Used video-conferencing tools?	◯	◯	◯	◯
Prepared an abstract for submission to a conference?	◯	◯	◯	◯
Prepared a manuscript for publication in a scientific journal?	◯	◯	◯	◯
Supervised staff?	◯	◯	◯	◯
Led an epidemiological investigation?	◯	◯	◯	◯
Led a response team or a control centre?	◯	◯	◯	◯
Managed a team?	◯	◯	◯	◯
Planned a project related to surveillance system development or implementation?	◯	◯	◯	◯

Any comments:

____

17. How many times have you participated in leadership and communication activities in the past 3 years?

◯ Never
◯ 1 - 2 times
◯ 3 -6 times
◯ More than 6 times

18. Based on the skills and experiences of the animal workers in your LGA, what priority would you say there is for further training in leadership and communication at the moment?

◯ No need for further training
◯ Low priority
◯ Moderate priority
◯ High priority
◯ Very high priority

Any comments:

____

Use of biosafety and biosecurity methods

19. How often have you used personal protective equipment (PPE - such as gloves, boots, mask and gowns) for handling sick animals during the last year?

	Never	Rarely	Sometimes	Always
Used gloves?	◯	◯	◯	◯
Used gumboots?	◯	◯	◯	◯
Used a surgical mask?	◯	◯	◯	◯
Used overalls/gown?	◯	◯	◯	◯
Used safety goggles/face shield?	◯	◯	◯	◯
Used P2 or N95 respirators?	◯	◯	◯	◯
Disposed off infectious materials?	◯	◯	◯	◯

Any comments:

____

20. How many times have you visited a farm in the past year?

____

21. When visiting farms, how often have you used the following biosecurity methods during the last year?

	Never	Rarely	Sometimes	Always
Cleaned boots before and after visiting a farm?	◯	◯	◯	◯
Washed hands with soap and water before and after visiting a farm?	◯	◯	◯	◯
Cleaned your vehicle before and after visiting a farm?	◯	◯	◯	◯
Disinfected your equipment before and after visiting a farm ?	◯	◯	◯	◯

Any comments?

____

22. Based on the skills and experiences of the animal workers in your LGA, what priority would you say there is for further training in biosafety and biosecurity methods at the moment?

- ◯ No need for further training
- ◯ Low priority
- ◯ Moderate priority
- ◯ High priority
- ◯ Very high priority

Any comments:

____

23. What is your age?

- ◯ Under 18
- ◯ 18-24
- ◯ 25-34
- ◯ 35-44
- ◯ 45-54
- ◯ 55-64
- ◯ 65+

24. What is your gender?

- ◯ Female
- ◯ Male
- ◯ Other

Other? __________________________

25. What is your work area?

Region __________________________

District __________________________

Township __________________________

Village __________________________

Postcode __________________________

26. What is your current position?

- ◯ Government veterinarian
- ◯ Township veterinary officer
- ◯ Deputy township veterinary officer
- ◯ Para-veterinarian or blue-cross worker
- ◯ Public Health officer
- ◯ Other (Specify)

Other (Specify): __________________________

27. What is your highest level of education in veterinary science?

- ◯ Diploma
- ◯ Bachelors
- ◯ Masters
- ◯ Doctorate
- ◯ Other(please specify)

Other (please specify) __________________________

28. In which year did you complete that education? __________________________

29. About how long have you been in your current position (years)? __________________________

30. Have you received any formal epidemiology training other than in your veterinary degree (select all that apply)?	☐ No<br>☐ Attended epidemiology workshops<br>☐ Enrolled in or completed a field epidemiology training programme<br>☐ Enrolled in or completed a postgraduate qualification in epidemiology<br>☐ Other (please specify) or Specify the level of epidemiology training undertaken
Other (please specify) or Specify the level of epidemiology training undertaken	__________________________________

Thank you for completing the survey! Your input will be valuable for our project.
