# Supplementary material for: Capacity and needs assessment of veterinary services in Vietnam in biosecurity, biosafety and One Health
Source: PLoS One. 2024 Jan 11;19(1):e0295898. doi: 10.1371/journal.pone.0295898 (PMC10783714; doi:10.1371/journal.pone.0295898)
Supplement: S2 Appendix — (DOCX) [file pone.0295898.s002.docx]

**Supplementary materials**

**Additional results from descriptive and univariable analysis**

**S1 Table.** One Health practices performed by respondents of the online survey conducted (n=178).

| How often have you done the following  in the last year? | **n (%)** | | | |
| --- | --- | --- | --- | --- |
|  | **Never** | **Rarely** | **About once a month** | **More than once a month** |
| Developed a control program for a zoonotic disease | 78 (45.6) | 64 (37.4) | 24 (14.0) | 5 (2.9) |
| Assisted in or led the investigation of a zoonotic disease | 48 (28.2) | 89 (52.4) | 26 (15.3) | 7 (4.1) |
| Assisted in or led the investigation of a non-zoonotic human disease | 48 (28.2) | 86 (50.6) | 27 (15.9) | 9 (5.3) |
| Participated in a team involving professionals from animals, human, and/or environmental sectors | 67 (39.6) | 78 (46.2) | 15 (8.9) | 9 (5.3) |

**S2 Table**. Univariable analyses results for level of experience in One Health (n=178). (Significant variables bolded)

| **Level of experience in One Health** | | | | | | |
| --- | --- | --- | --- | --- | --- | --- |
| **Variable**  **(Demographic characteristics)** | **Categories** | **Estimate** | **SE** | **Odds ratio** | **95% CI of odds ratio** | **P-value** |
| **Age** | 18-34^1^ | 0.00 |  | 1.00 |  | **0.02** |
|  | 35-44 | 1.20 | 0.48 | 3.31 | (1.36, 9.34) |  |
|  | ≥45 | 1.15 | 0.65 | 3.14 | (0.86, 11.65) |  |
| **Gender** | Female^1^ | 0.00 |  | 1.00 |  | 0.8 |
|  | Male | -0.07 | 0.35 | 0.93 | (0.46, 1.84) |  |
| **Work role** | District veterinary officer^1^ | 0.00 |  | 1.00 |  | **0.009** |
|  | Provincial veterinary officer | 1.19 | 0.39 | 3.29 | (1.54, 7.26) |  |
|  | Other | 0.68 | 0.66 | 1.97 | (0.49, 6.88) |  |
| **Education level** | Bachelor^1^ | 0.00 |  | 1.00 |  | **0.07** |
|  | Diploma or other | -0.55 | 0.80 | 0.58 | (0.09, 2.31) |  |
|  | Postgraduate | 0.76 | 0.38 | 2.15 | (1.02, 4.52) |  |
| **Have you completed any formal epidemiology training?** | No formal training completed^1^ | 0.00 |  | 1.00 |  | **0.02** |
|  | Formal training completed | 0.89 | 0.38 | 2.44 | (1.18, 5.30) |  |
|  | No epidemiology workshops attended^1^ | 0.00 |  | 1.00 |  | **0.004** |
|  | Attended epidemiology workshops | 0.61 | 0.35 | 1.83 | (0.92, 3.68) |  |
|  | No postgraduate qualification or FETP^1^ | 0.00 |  | 1.00 |  | **0.003** |
|  | Postgraduate qualification or FETP | 1.43 | 0.46 | 4.19 | (1.69, 10.53) |  |
| **Job tenure** | 0-9^1^ | 0.00 |  | 1.00 |  | **0.002** |
|  | 10-12 | 2.21 | 0.60 | 9.16 | (2.84, 29.53) |  |
|  | ≥13 | 2.32 | 0.59 | 10.14 | (3.21, 31.99) |  |

| How often have you used PPE for handling sick animals during the last year? | **n (%)** | | | |
| --- | --- | --- | --- | --- |
|  | **Never** | **Rarely** | **About once a month** | **More than once a month** |
| Used gloves | 0 (0) | 4 (2.3) | 16 (9.4) | 151 (88.3) |
| Used gumboots | 1 (0.6) | 8 (4.7) | 58 (34.1) | 103 (60.6) |
| Used a surgical mask | 4 (2.4) | 4 (2.4) | 19 (11.1) | 143 (84.1) |
| Used overalls/gown | 4 (2.3) | 19 (11.1) | 44 (25.7) | 104 (60.8) |
| Used safety goggles/face shield | 9 (5.3) | 29 (17.0) | 63 (36.8) | 70 (40.9) |
| Used P2 or N95 respirators | 24 (14.0) | 47 (27.5) | 43 (25.1) | 57 (33.3) |
| Disposed of infectious materials | 16 (9.5) | 23 (13.6) | 34 (20.1) | 96 (56.8) |

**S3 Table.** Use of biosafety materials by the survey respondents in Vietnam (n=178).

**S4 Table.** Use of biosafety materials and biosecurity practices by the survey respondents in Vietnam (n=178).

| When visiting farms, how often have you used the following biosecurity methods during  the last year? | **n (%)** | | | |
| --- | --- | --- | --- | --- |
|  | **Never** | **Rarely** | **About once a month** | **More than once a month** |
| Cleaned boots before and after visiting a farm | 0 (0) | 1 (0.6) | 10 (5.9) | 158 (93.5) |
| Washed hands with soap and water before and after visiting a farm | 0 (0) | 1 (0.6) | 11 (6.6) | 155 (92.8) |
| Cleaned your vehicle before and after visiting a farm | 2 (1.2) | 11 (6.6) | 33 (19.8) | 121 (72.4) |
| Disinfected your equipment before and after visiting a farm | 0 (0) | 4 (2.4) | 17 (10.0) | 148 (87.6) |

**Table S5**. Univariable analyses results showing the level of experience in biosafety and biosecurity (n=178). (Significant variables bolded)

| **Level of experience in biosafety** | | | | | | |
| --- | --- | --- | --- | --- | --- | --- |
| **Variable**  **(Demographic characteristics)** | **Categories** | **Estimate** | **SE** | **Odds ratio** | **95% CI of odds ratio** | **P-value** |
| **Age** | 18-34^1^ | 0.00 |  | 1.00 |  | 0.39 |
|  | 35-44 | 0.44 | 0.32 | 1.55 | (0.82, 2.94) |  |
|  | ≥45 | 0.39 | 0.49 | 1.48 | (0.56, 3.87) |  |
| **Gender** | Female^1^ | 0.00 |  | 1.00 |  | 0.61 |
|  | Male | 0.14 | 0.28 | 1.15 | (0.66, 2.02) |  |
| **Work role** | District veterinary officer^1^ | 0.00 |  | 1.00 |  | 0.60 |
|  | Provincial veterinary officer | 0.29 | 0.32 | 1.34 | (0.72, 2.49) |  |
|  | Other | 0.35 | 0.55 | 1.42 | (0.48, 4.20) |  |
| **Education level** | Bachelor^1^ | 0.00 |  | 1.00 |  | 0.29 |
|  | Diploma or other | 0.87 | 0.55 | 2.38 | (0.80, 7.20) |  |
|  | Postgraduate | 0.06 | 0.33 | 1.06 | (0.56, 2.00) |  |
| **Have you completed any formal epidemiology training?** | No formal training completed^1^ | 0.00 |  | 1.00 |  | 0.36 |
|  | Formal training completed | 0.26 | 0.29 | 1.30 | (0.71, 2.12) |  |
|  | No epidemiology workshops attended^1^ | 0.00 |  | 1.00 |  | 0.47 |
|  | Attended epidemiology workshops | 0.20 | 0.28 | 1.22 | (0.71, 2.12) |  |
|  | No postgraduate qualification or FETP^1^ | 0.00 |  | 1.00 |  | 0.28 |
|  | Postgraduate qualification or FETP | 0.45 | 0.41 | 1.57 | (0.70, 3.54) |  |
| **Job tenure** | 0-9^1^ | 0.00 | 0.03 | 1.00 |  | **0.015** |
|  | 10-12 | 0.95 | 0.41 | 2.59 | (1.18, 5.87) |  |
|  | ≥13 | 0.96 | 0.39 | 2.61 | (2.61, 1.23) |  |
| **Level of experience in biosecurity** | | | | | | |
| **Variable**  **(Demographic characteristics)** | **Categories** | **Estimate** | **SE** | **Odds ratio** | **95% CI of odds ratio** | **P-value** |
| **Age** | 18-34^1^ | 0.00 |  | 1.00 |  | 0.30 |
|  | 35-44 | -0.27 | 0.37 | 0.76 | (0.36, 1.56) |  |
|  | ≥45 | -0.85 | 0.54 | 0.43 | (0.15, 1.24) |  |
| **Gender** | Female^1^ | 0.00 |  | 1.00 |  | 0.35 |
|  | Male | -0.30 | 0.32 | 0.74 | (0.40, 1.38) |  |
| **Work role** | District veterinary officer^1^ | 0.00 |  | 1.00 |  | 0.44 |
|  | Provincial veterinary officer | 0.15 | 0.35 | 1.17 | (0.59, 2.34) |  |
|  | Other | 0.83 | 0.69 | 2.30 | (0.66, 10.74) |  |
| **Education level** | Bachelor^1^ | 0.00 |  | 1.00 |  | **0.12** |
|  | Diploma or other | 1.20 | 0.79 | 3.30 | (0.84, 21.95) |  |
|  | Postgraduate | -0.30 | 0.36 | 0.74 | (0.37, 1.51) |  |
| **Have you completed any formal epidemiology training?** | No formal training completed^1^ | 0.00 |  | 1.00 |  | 0.85 |
|  | Formal training completed | -0.06 | 0.31 | 0.94 | (0.51, 1.74) |  |
|  | No epidemiology workshops attended^1^ | 0.00 |  | 1.00 |  | 0.35 |
|  | Attended epidemiology workshops | 0.29 | 0.31 | 1.34 | (0.73, 2.50) |  |
|  | No postgraduate qualification or FETP^1^ | 0.00 |  | 1.00 |  | 0.87 |
|  | Postgraduate qualification or FETP | -0.07 | 0.46 | 0.93 | (0.38, 2.36) |  |
| **Job tenure** | 0-9^1^ | 0.00 |  | 1.00 |  | **0.11** |
|  | 10-12 | 0.83 | 0.43 | 2.30 | (1.01, 5.49) |  |
|  | ≥13 | 0.10 | 0.38 | 1.10 | (0.53, 2.33) |  |
